# B-Cell Epitopes in NTS-DBL1α of PfEMP1 Recognized by Human Antibodies in Rosetting *Plasmodium falciparum*

**DOI:** 10.1371/journal.pone.0113248

**Published:** 2014-12-01

**Authors:** Letusa Albrecht, Davide Angeletti, Kirsten Moll, Karin Blomqvist, Davide Valentini, Fabio Luiz D'Alexandri, Markus Maurer, Mats Wahlgren

**Affiliations:** 1 Department of Microbiology, Tumor- and Cell Biology (MTC), Karolinska Institutet, Stockholm, Sweden; 2 Therapeutic Immunology (TIM), Department of Laboratory Medicine, Karolinska Institutet, Stockholm, Sweden; 3 CAST, Karolinska University Hospital, Huddinge, Sweden; 4 Department of Biochemistry, University of Campinas, Campinas, Brazil; London School of Hygiene and Tropical Medicine, United Kingdom

## Abstract

*Plasmodium falciparum* is the most lethal of the human malaria parasites. The virulence is associated with the capacity of the infected red blood cell (iRBC) to sequester inside the deep microvasculature where it may cause obstruction of the blood-flow when binding is excessive. Rosetting, the adherence of the iRBC to uninfected erythrocytes, has been found associated with severe malaria and found to be mediated by the NTS-DBL1α-domain of *Plasmodium falciparum* Erythrocyte Membrane Protein 1 (PfEMP1). Here we show that the reactivity of plasma of Cameroonian children with the surface of the FCR3S1.2-iRBC correlated with the capacity to disrupt rosettes and with the antibody reactivity with a recombinant PfEMP1 (NTS-DBL1α of IT4_var60_) expressed by parasite FCR3S1.2. The plasma-reactivity in a microarray, consisting of 96 overlapping 15-mer long peptides covering the NTS-DBL1α domain from IT4var60 sequence, was compared with their capacity to disrupt rosettes and we identified five peptides where the reactivity were correlated. Three of the peptides were localized in subdomain-1 and 2. The other two peptide-sequences were localized in the NTS-domain and in subdomain-3. Further, principal component analysis and orthogonal partial least square analysis generated a model that supported these findings. In conclusion, human antibody reactivity with short linear-peptides of NTS-DBL1α of PfEMP1 suggests subdomains 1 and 2 to hold anti-rosetting epitopes recognized by anti-rosetting antibodies. The data suggest rosetting to be mediated by the variable areas of PfEMP1 but also to involve structurally relatively conserved areas of the molecule that may induce biologically active antibodies.

## Introduction

Malaria is the most important of all parasitic diseases. About 200 million people are affected by malaria infections and 1.44 billion people worldwide are at risk of *Plasmodium falciparum* malaria. Malaria particularly affects children under the age of 5 and women in their first pregnancy in endemic areas [1].

It is known that repeated exposure to *P. falciparum* parasites induces immunity to severe disease. This protective immunity is partly dependent on antibodies towards variable surface proteins expressed by the parasite blood stages, where *Plasmodium falciparum* Erythrocyte Membrane Protein 1 – PfEMP1 is one of the major antigens [2]. PfEMP1 also plays a central role in the ability of the parasite to sequester in the microvasculature of the infected patient. It mediates binding to a variety of different host-cell receptors enabling the iRBC to sequester in the deep microvasculature in order to avoid clearance in the spleen. PfEMP1 contributes substantially to the manifestations of severe malaria as sequestration becomes excessive and blocks the blood flow.

A central feature of *P. falciparum* is the ability to cytoadhere to various host receptors on different cell types and serum proteins. One important adhesive phenotype, associated with disease severity, is the formation of rosettes, where an infected erythrocyte (iRBC) adheres to two or more non-infected red cells, RBC [3],[4],[5],[6],[7]. The ability to form rosettes varies in-between *P. falciparum* strains and a range of host cell receptors on the surface of RBC as well as serum-proteins are involved in the binding phenomena. These include heparan sulfate, complement receptor CD35, blood group A and B trisaccharides and maybe CD36 as well as immunoglobulins M and G, fibrinogen and albumin [8],[9]. PfEMP1 mediates the binding and antibodies towards this protein can disrupt rosettes [10],[11],[12],[13],[14]. For laboratory parasites of a rosetting phenotype such as FCR3S1.2, varO and R29, the N-terminal Duffy-binding like domain (DBL1α) has been shown to be the key domain of the PfEMP1 molecule binding to host receptors on RBC [15],[16],[17],[18]. This domain has the highest degree of sequence conservation among all PfEMP1 domains [19] and is therefore likely to hold a central role in parasite sequestration in the microvasculature [17],[20],[21].

A large fraction of immunity towards severe disease is conferred by antibody responses to PfEMP1 [22], as a consequence of the central role it holds in sequestration, but it is not understood how immunity to this highly variable antigen develops. Clinical data suggest that patients rapidly acquire immunity that protects against severe disease [23],[24],[25],[26]. One possible scenario is that protection is achieved after acquiring cross-reactive, strain-transcending antibodies to a few conserved epitopes shared among several PfEMP1 variants. On the other hand, immunity could also rely on a large pool of strain-specific antibodies acquired over time. Indeed, varying degrees of serological cross reactivity have been demonstrated by studying sera from malaria infected individuals or sera from PfEMP1-immunized animals on heterologous PfEMP1 proteins [27],[28],[29],[30],[31]. Epitopes recognized by cross-reactive antibodies are largely unknown. Recently one such epitope was identified when immunization with a PfEMP1 motif associated with severe malaria generated strain transcending antibodies, however the function of the generated antibodies remains unknown [32]. There is still a lack of information about antibodies acquired during natural malaria infections, their potential cross-reactivity and their role in immunity against severe malaria. Here, we show that the ability of antibodies to disrupt rosettes is correlated with the presence of particular antibody-specificities. When the binding pattern of naturally acquired antibodies was mapped on a peptide array, the reactivity towards five peptides of the NTS-DBL1α-domain correlated with the capacity of the same plasma samples to disrupt rosettes of the homologous parasite (FCR3S1.2). The reactive peptides were localized within all three subdomains of the NTS-DBL1α-domain. Further, global analysis of the plasma reactivity in the array suggested that presence of rosette disruptive antibodies correlated with recognition of the semi-conserved region at the C-terminus of subdomain 2 and N-terminus of subdomain 3. The results suggest that, in spite of PfEMP1 variability, some of the antibodies directed towards this molecule are likely to react with conserved epitopes.

## Results

### Rosette disruption activity and surface reactivity

**Figure 1:**
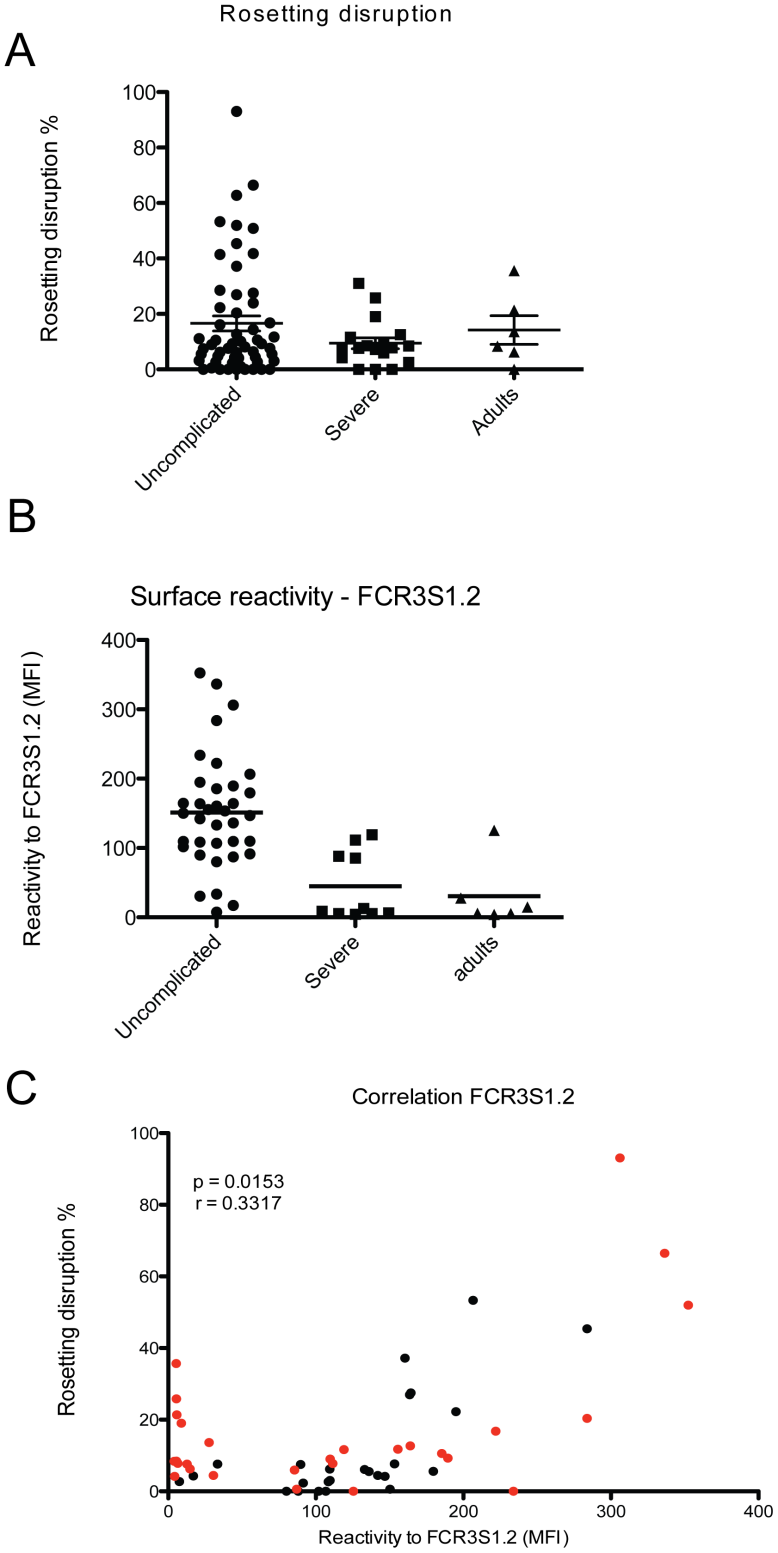
Individuals living in malaria endemic areas carry antibodies able to disrupt rosettes. A) 75 plasma samples derived from patients living in endemic areas were tested for their capacity to disrupt rosettes of FCR3S1.2 iRBC. B) The plasma ability to bind to the surface of FCR3S1.2 iRBC was measured by flow cytometry. Uncomplicated and severe indicate malaria patients from Cameroon while Adults indicate individuals living in an area of lower endemicity (Yunnan province, China). C) Correlation between rosette disruption and surface reactivity on FCR3S1.2 iRBC for all patients samples. In red are indicated samples that are further analyzed for peptide recognition in the peptide array.

In order to identify antibodies that are able to target adhesive events, such as the formation of rosettes, we analyzed a set of plasma, derived from patients living in a malaria endemic area, for the presence of antibodies that are able to disrupt rosettes formed by the laboratory clone FCR3S1.2. This strain is considered as a model parasite since it displays a phenotype typical for parasites causing severe disease, with a high rosetting rate and the ability to bind non-immune IgM to its surface [33]. Seventy-five plasma samples of children between 6 months and 14 years of age, infected with *P. falciparum* from Buea, an endemic region of Cameroon, were investigated [34], (Table S1). Eighteen samples were from children with severe malaria and 57 from uncomplicated malaria. In this set of samples, the average age was 55.4 months; whereas the mean in the severe malaria group was 28.9 months in the uncomplicated group was 49.5 months (p = 0.0671). Out of 75 tested samples 29.3% (22 samples) were able to disrupt rosettes of FCR3S1.2. The cut off to be considered as positive in rosette disruption assays was defined as >15% disruption compared to the control [3]. Three samples or 13.6% of the 22 samples tested positive were from patients with severe malaria, which represents 16.7% of severe samples (3/18). The remaining 19 samples were from uncomplicated cases, representing 33.3% of uncomplicated tested samples (19/57) (Figure 1A). Further, plasma samples from uncomplicated malaria cases were able to disrupt rosettes by 16.3% in average, while samples from severe malaria patients resulted in an average reduction of 9.4%. Fifty-three samples did not have ability to disrupt rosettes, which represents 70.7% of analyzed samples, whereas 71.7% were from uncomplicated cases and the remaining 28.3% (15 samples) were from severe ones. However, there was no statistical difference between severe and uncomplicated groups (p value  = 0.6047). As comparison, two out of six samples from adults living in a low endemic area tested positive for rosette disruption and the average of rosette disruption was 14.2%.

The surface reactivity on FCR3S1.2 iRBC with the same plasma samples was tested in parallel. The cut-off level for positivity was set to 83.6 AU, based on non-immune plasma reactivity. Surface reactive antibodies were detected in 63.3% of the samples. In details, 83.6% of the samples from the uncomplicated group and 37.5% from the severe group gave a positive surface staining (p value <0.0001). In addition, the average staining intensity of the samples belonging to the uncomplicated malaria group was three times higher as compared to samples belonging to the severe malaria group (p value  = 0.0002; Figure 1B). There was no difference between the group with severe malaria and a group of adults residing in low endemic area (mean of 50.2 and 30.5 respectively). Interestingly, a positive correlation was found between surface reactivity and ability to disrupt rosettes of the plasma (Figure 1C), suggesting that the same subset of molecules might be targeted in the two assays. The same dataset was also analyzed as a contingency table (2×2 table), where samples were considered positive or negative for surface recognition and rosetting disruption. Fischer's exact test was applied and a p value <0.0001 supports the data described above.

### Seroprevalence of human anti-NTS-DBL1α antibodies

The parasite strain FCR3S1.2 displays a PfEMP1 that is encoded by the IT4_var60_ gene on the surface of which the NTS-DBL1α domain has been demonstrated to mediate the binding to other RBCs [13]. As the plasma samples from Cameroonian children tested positive for rosette disruption and surface reactivity with the FCR3S1.2 strain, we investigated, as a next step, the presence of antibodies towards NTS-DBL1α-IT4_var60_ using ELISA assays.

**Figure 2:**
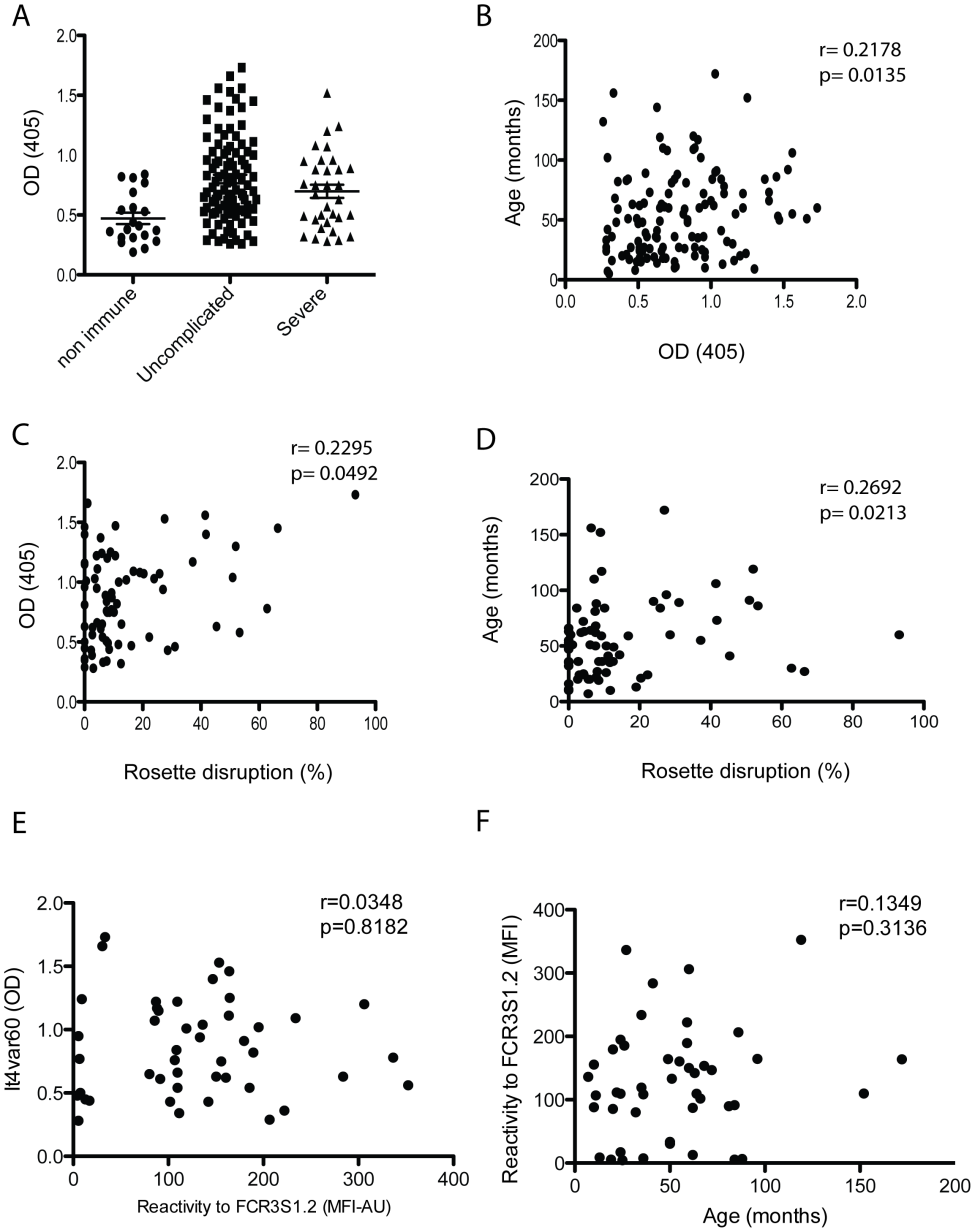
Naturally acquired antibodies to NTS-DBL1α. A) IgG levels in 134 human plasma samples against the recombinant NTS-DBL1α^It4var60^ as detected by ELISA. Non immune indicates 20 non immune Swedish donors. Uncomplicated and severe indicate patients from Cameroon. B) Correlation between patient age and presence of antibodies in the plasma towards the recombinant NTS-DBL1α^It4var60^ as measured by ELISA. C) Correlation between ability of the plasma sample to disrupt FCR3S1.2 iRBC rosettes and presence of antibodies towards to NTS-DBL1α^It4var60^ as measured by ELISA. D) Correlation between ability of the sample to disrupt FCR3S1.2 iRBC rosettes and patient age. E) Correlation between ability of the plasma sample to recognize FCR3S1.2 iRBC surface as detected by FACS and presence of antibodies towards to NTS-DBL1α^It4var60^ as measured by ELISA. F) Correlation between ability of the plasma sample to recognize FCR3S1.2 iRBC surface as detected by FACS and patient age.

The 75 samples used for the rosette disruption and surface reactivity assays plus other 59 samples were used for ELISA. A total of 134 samples of children of 6 months to 14 years of age were analyzed; 101 were from children with uncomplicated and 33 from children with severe malaria, including severe anemia, cerebral malaria and respiratory distress. Antibodies towards NTS-DBL1α-IT4_var60_ were detected in 44 plasma samples (32.8%), with the majority of positive samples belonging to the uncomplicated malaria group. In the uncomplicated malaria group 36 samples had specific antibodies to NTS-DBL1α IT4_var60_ (35.6%) while in the severe malaria group 8 samples (24.2%) had antibodies towards NTS-DBL1α-IT4_var60_ (Figure 2A). No statistical difference was observed between those groups (p = 0.1578). The presence of antibodies was positively correlated with age of the children (Spearman r = 0.2178, p value  = 0.0135; Figure 2B). A positive correlation was also found between the presence of naturally acquired antibodies to NTS-DBL1α-IT4_var60_ and the ability to disrupt rosettes (Spearman r = 0.2295, p = 0.0492; Figure 2C). Further, the ability to disrupt rosettes of the FCR3S1.2 strain correlated with age (r = 0.2692 and p = 0.0213) (Figure 2D). However, there was no correlation between the surface reactivity and presence of antibodies towards to IT4_var60_ (Figure 2E) or age (Figure 2F).

### Analysis of clinical and immunological data

The clinical features as well as the immunological data of the Cameroonian population were studied also analyzed using Principal Component Analysis – PCA. This approach is an unsupervised method that gives an overall trend about the data and inspection about outliers. The parameter used for this analysis is the R2, which explains the variation of the data and gives the predictability of the model. The program generated a proposed model that can explain 23% of the data.

**Figure 3:**
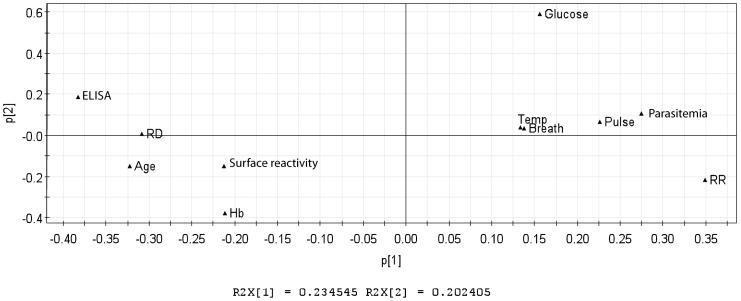
Principal Component Analysis of clinical and immunological data. Data analysis from Cameroonian samples where clinical parameters and immunological data were analyzed using Principal Component Analysis. The principal component analysis generated a model that explains 23% of the data segregation (R2X  = 0.234545). Clinical parameters: temperature (Temp), breath rate (breath), pulse rate (pulse), glucose level (glucose), haemoglobin (Hb), parasitemia, age, rosetting rates (RR, after collection from patients). Immunological parameters: plasma sample recognition of NTS-DBL1α It4var60 domain (ELISA), ability to disrupt FCR3S1.2 rosettes (RD) and ability to recognize FCR3S1.2 iRBC surface by flow cytometry (Surface reactivity).

This analysis suggests that the presence of antibodies towards to NTS-DBL1α-IT4_var60_ was inversely linked with parasitemia in the peripheral blood, which is reinforced when correlation by Spearman test was analyzed (r = −0.4943, p<0.0001). Further, the presence of rosettes in the peripheral blood was inversely correlated with the presence of specific antibodies to NTS-DBL1α-IT4_var60_ (Figure 3). The capacity to disrupt rosettes in the Cameroonian samples was positively correlated both to the presence of antibodies to IT4_var60_ and the age of the children, which suggests that older children have higher levels of antibodies compared to the younger ones, with an augmented capacity to disrupt rosettes. Although the model explains 23% of the data, some other parameters that are not included in this analysis could be involved in the ability to disrupt rosettes, such as the presence of antibodies to other surface antigens.

### Identification of B- cell epitopes important for rosetting

**Figure 4:**
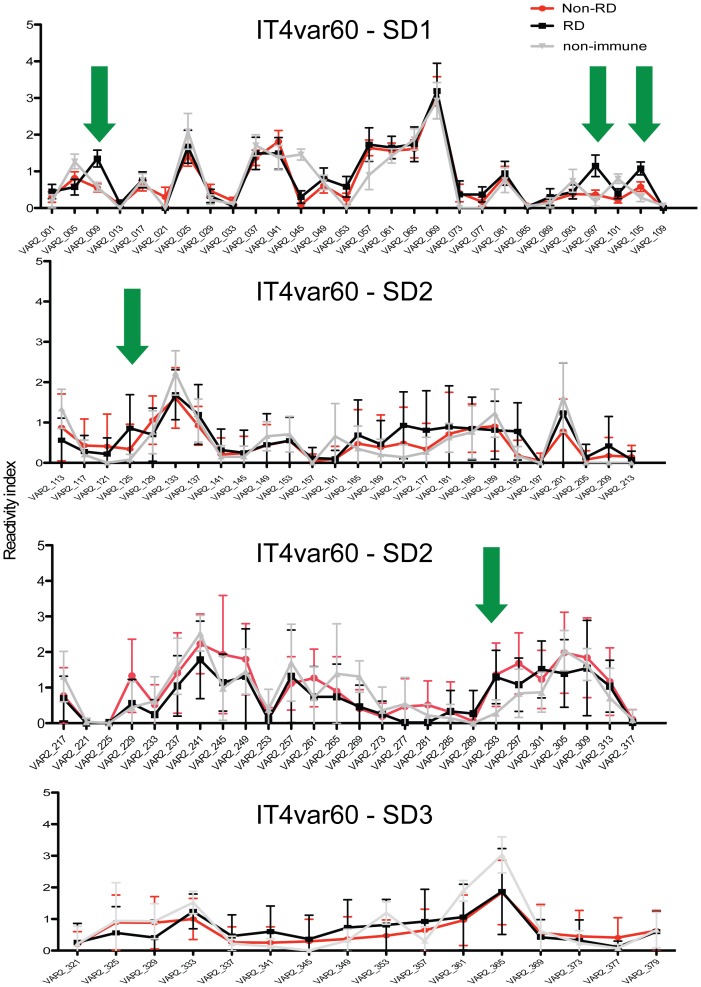
Reactivity of human plasma to a peptide array of the NTS-DBL1α^It4var60^. A subset of 26 plasma samples (red dots in figure 1C; further described in Table 1) were tested on a peptide microarray covering the NTS-DBL1α^It4var60^ domain. 9 plasma samples were able to disrupt FCR3S1.2 iRBC rosettes while 17 were not. The mean value for the reactivity index of each sample group was compared to the mean reactivity index of 8 non-immune control samples. Green arrows indicate peptides with differential recognition between the groups.

**Figure 5:**
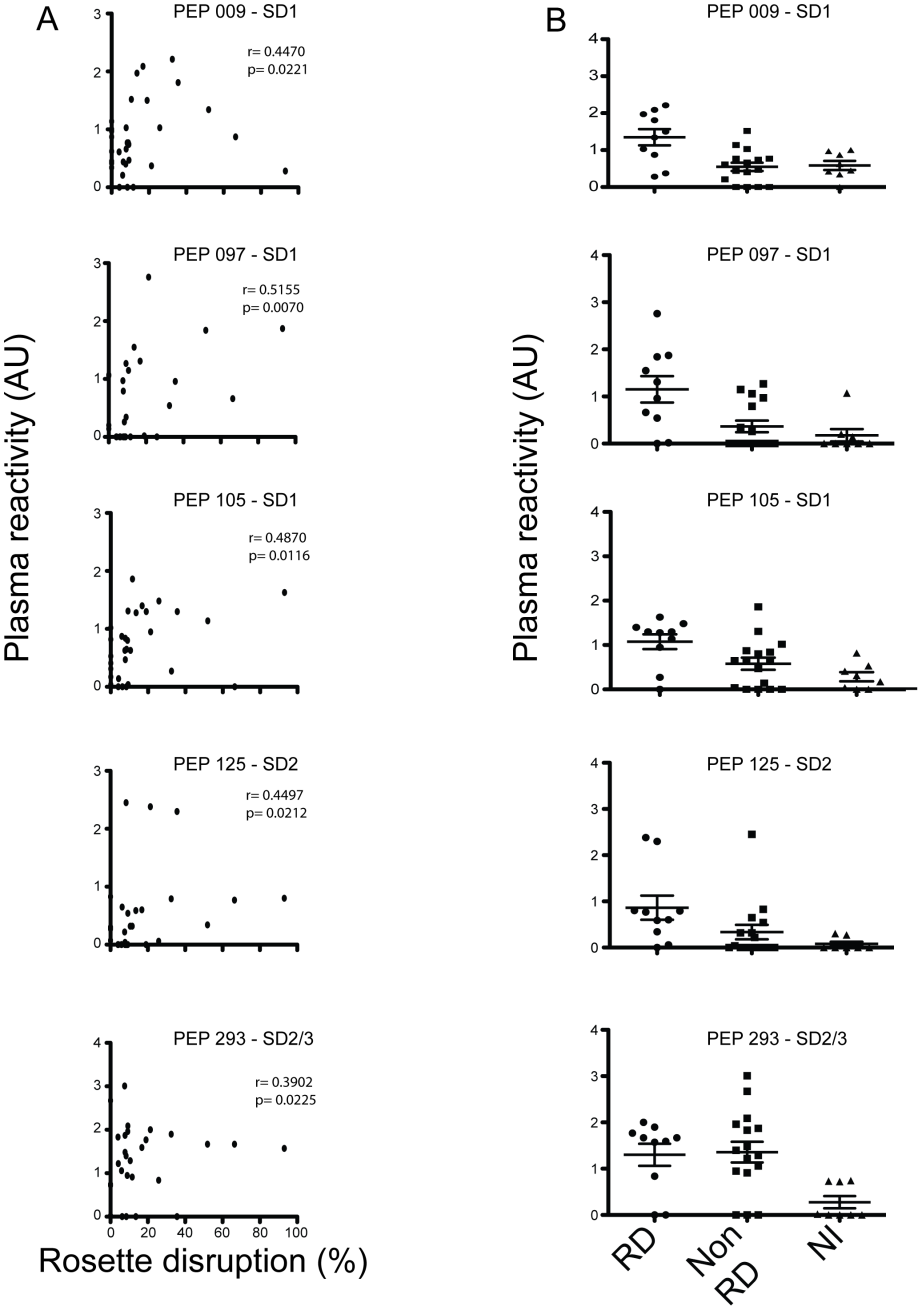
Peptides associated with the ability to disrupt rosettes of the FCR3S1.2 strain. Reactivity towards five peptides (indicated by green arrows in figure 4) was correlated with plasma sample ability to disrupt FCR3S1.2 rosettes. A) Scatter plots showing the correlation between specific peptide recognition and the ability to disrupt rosettes of FCR3S1.2 iRBC. B) Reactivity index measured in peptide array to the same peptides according to their grouping: rosette disruptive (RD), non-rosette disruptive (Non-RD) and Swedish non-immune control samples (NI).

The identification of epitopes associated with disruption of rosettes is of importance for the development of a vaccine that prevents rosetting and protects against severe disease. To discover potential B-cell epitopes targeted by rosette disruptive antibodies, the reactivity of a subset of 26 plasma samples was investigated in a peptide array (Table 1). Plasma samples were divided in two groups according to their ability to disrupt rosettes (rosette disruptive, RD, n = 9; and non-rosettes disruptive samples, Non-RD, n = 17) and their reactivity on the peptide array was compared. The array comprises overlapping peptides covering the entire NTS-DBL1α-IT4_var60_ (Figure 4). The reactivity of the sera with five out of the 96 peptides, here named PEP_009, PEP_097, PEP_105, PEP_125, PEP_293, correlated with the ability to disrupt rosettes formed by FCR3S1.2 iRBCs (Figure 5A). One peptide was found at the N-terminus of the NTS domain (PEP_009; Spearman r = 0.4470, p value  = 0.0221) while peptides PEP_097, PEP_105 and PEP_125 were spanning a sequence located at the N-terminal on the DBL1α domain (PEP_097, Spearman r = 0.5155, p value  = 0.0070; PEP_105, Spearman r = 0.4870 and p value  = 0.0116, PEP_125; Spearman r = 0.4497, p value  = 0.0212). The peptide PEP_293 was found in subdomain 3 in the boundary to subdomain 2 (PEP_293, Spearman r = 0.3902, p value  = 0.0225). The reactivity of the non-immune plasma with these five peptides was negative or at background level (Figure 5B).

**Table 1:** Peptide array sample groups.

	Surface reactivity to FCR3S1.2 (MFI)	ELISA reactivity to NTS-DBL1α (It4var60) (OD)	Rosette Disruption in FCR3S1.2 (%)
NR_CBS08	88.125	0.96	0.00
NR_CBM20	30.6	1.47	4.42
NR_CBM31	109.725	1.25	8.99
NR_CBS35	111.45	1.20	7.78
NR_CBS47	6.5	0.75	7.8
NR_CBS60	12.7	0.76	7.59
NR_CBS89	4.4	0.95	4.2
NR_CBS91	5.5	0.44	8.45
NR_CBM75	185.385	1.22	10.57
NR_CBM77	87.06	1.03	0.62
NR_CBS88	119.08	0.48	11.63
NR_CBM46	189.515	0.77	9.26
NR_CBS162	85.62	1.24	5.94
NR_BUR04	4.07	0.00	8.37
NR_BUR10	125.45	0.18	0.00
NR_BUR11	14.70	0.00	6.26
NR_BUR03	27.71	0.00	13.61
RD_CBM36	306.13	1.73	93.08
RD_CBM41	352.39	1.30	51.97
RD_CBM43	283.81	1.07	20.35
RD_CBM54	222	1.09	16.79
RD_CBM68	336.45	1.45	66.44
RD_CBS155	5.495	1.07	25.83
RD_CBS168	8.895	1.08	19.02
RD_BUR07	5.47	0.42	35.66
RD_BUR08	5.84	1.08	21.38

Summary of the subset of samples further analyzed on the peptide array (shown as red dots in Figure 1C). The table summarizes the sample name (NR: non rosette disruptive; RD: rosette disruptive), surface reactivity towards FCR3S1.2 iRBC (MFI), the ELISA reactivity towards the NTS-DBL1α domain and ability to disrupt the FCR3S1.2 iRBCs rosettes.

### Identification of epitopes recognized by rosette inhibitory antibodies

**Figure 6:**
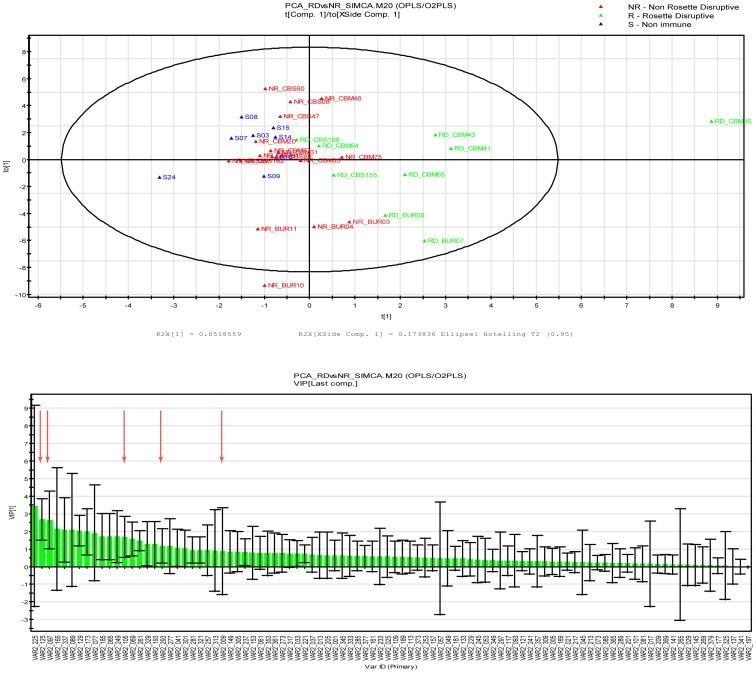
Orthogonal Partial Least Square analysis (OPLS) of peptide array data for It4var60. Distribution of samples on the OPLS model according to their reactivity on the peptide array (top). VIP ranking score for the OPLS analysis (Bottom). Red arrows indicate the peptides correlated with the ability to disrupt rosettes as seen in figure 5.

In order to confirm the data obtained and to better correlate the identified antibody epitopes to the rosette disruptive capacity of the human samples, a multivariate statistical analysis, taking into account all parameters, was performed. Principal component analysis (PCA) was followed by orthogonal partial least square (OPLS) analysis that is a supervised method and gives the data segregation between classes along the predicted components. Two parameters were used to judge the model, the R2 and the Q2, where R2 explains the variation of the data and the Q2 is a cross validation factor that gives the predictability of the model. Data obtained from testing reactivity of plasma on the NTS-DBL1α-IT_var60_ peptide array in combination with the information about their ability to disrupt rosettes on the FCR3S1.2 strain were analyzed. The model generated can explain 20% of the data segregation, with the “rosette disruptive group” being well separated from the “non-rosette disruptive” and the “non-immune group” (Figure 6). In this analysis, the majority of peptides were not informative (68%) since their reactivity was not different between the groups, but all five of the previously identified peptides where the plasma reactivity was correlated to the ability to disrupt rosettes, were among the top 30 of the 96 peptides ranked in this segregation (Figure 6).

### Epitopes targeted by rosette disrupting antibodies within the NTS-DBL1α-IT4_var60_ domain

**Figure 7:**
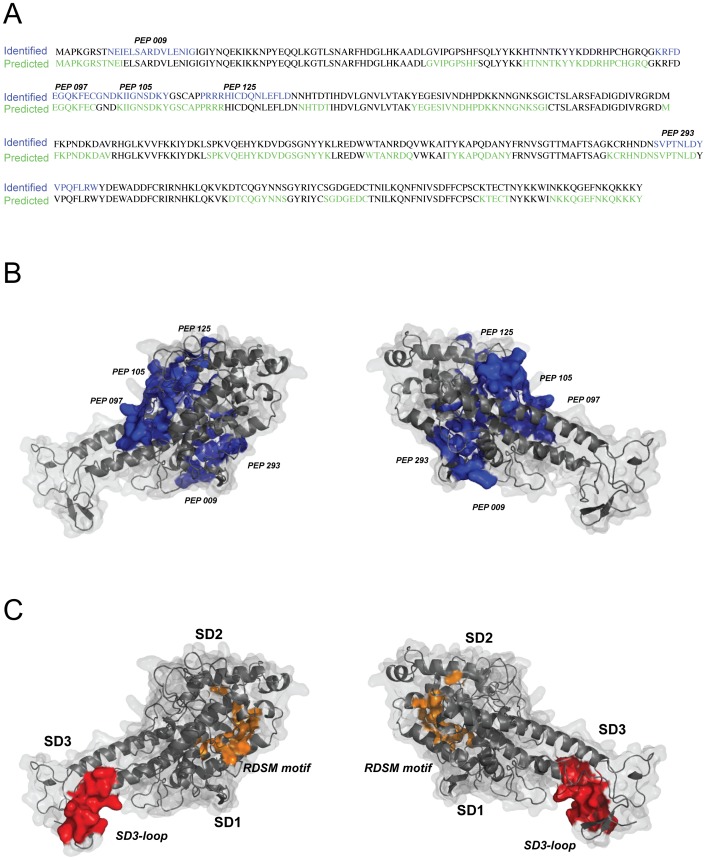
Localization of epitopes targeted by rosette disruptive on the NTS-DBL1α^It4var60^ domain. A) Comparison between predicted epitopes (predicted, in green) and epitopes correlated with rosette disruption ability (identified, in blue). Prediction of the localization of B-cell epitopes for the NTS-DBL1α^It4var60^ domain was carried out and epitopes predicted to be surface exposed are marked in green. Epitopes that correlated with the ability to disrupt rosettes are marked in blue. B) A model of the NTS-DBL1α^It4var60^ domain was generated based on the NTS-DBL1α^PAvarO^ crystal structure [18]. The mapped peptides targeted by antibodies that disrupted rosettes on FCR3S1.2 iRBC are highlighted in blue in the model. C) For comparison, the localization SD3-loop, previously identified as target of rosette disruptive antibodies (12) is highlighted in red and the RDSM motif, shown to induce strain-transcending antibodies [32], in orange.

Since the crystal structure of the NTS-DBL1α-IT4_var60_ molecule is not available, a molecular model was generated using the crystal structure of the homologous NTS-DBL1α from PAvarO. The five sequences identified with the peptide array were all found to be localized on the solvent accessible surface of the molecule (Figure 7B), with the exception of PEP_293 that was only partially exposed. While peptides PEP_097, PEP_105 and PEP125 were overlapping and found to be part of the same linear epitope located at the N-terminus of the DBL1α domain in SD1, peptides PEP_009 and PEP_293 were single linear epitopes. PEP_293 is in addition in close proximity with RDSM peptide previously described as target of strain transcending antibodies in SD2 [32]. By using an algorithm for the prediction of B-cell epitopes trained on previously experimentally proven epitopes called Bepipred [35], out of a total of 15 predicted B cells epitopes in the NTS-DBL1α domain, only part of the peptides were detected in this analysis (Figure 7A). Further, 20 out of 23 aa were suggested to be surface exposed in peptides PEP_097 and PEP_105 while the sequences of peptides PEP_125 and PEP_293 were predicted to be only partially exposed. The peptide PEP_293 contains part of a motif previously found to be conserved between different DBL1α sequences (PoLV4, ref [36]).

### The reactivity of the plasma with other DBL domains

The ability to disrupt rosettes of FCR3-parasites has been correlated with protection against severe forms of malaria [3]. FCR3S1.2 is a highly rosetting, IgM binding subclone expressing the same PfEMP1-encoding *var* gene (IT4_var60_) that belongs to DBLα1 group A, which has been found associated with severe malaria [37]. We therefore decided to classify our malaria samples based on the ability to disrupt rosettes formed by FCR3S1.2 1 and study their reactivity in a broad microarray screen harboring multiple NTS-DBL1α-domains. The two groups of plasma included 1) rosettes disruptive (RD)- and 2) non-disruptive (NR) samples (Table 1). A peptide array with 15 aa long-peptides with a 11 aa overlap covering the NTS-DBL1α of IT4_var60_ and 8 complementary DBL-domains were included in the array (Table S2). Three were NTS-DBL1α domains associated with rosetting in lab strains, whereas IT4_var9_ were from R29, TM284S2 was derived from TM284, and PA_varO_ which was derived from the lab strain Palo Alto [14],[15],[38]. Another sequence NTS-DBL1α associated with severe malaria - 3D7var4, was derived from the lab clone 3D7 [37]. Three DBL1α sequences expressed by parasites isolated from severe Ugandan patient samples (UAS22, UAS29 and UAS31 [7] and the NTS-DBL1α-IT4_var21_domain [13].

The ability to disrupt rosettes was positively correlated with 33 peptides. Five of these peptides were from NTS-DBL1α-IT4_var9_ (derived from R29), five from the NTS-DBL1α-TM284 and two from the NTS-DBL1α-PA_varO_ (Table 2). Interestingly, for all three parasites strains, at least one of the identified peptides (It4var9/PEP_293, TM284/PEP_293, PAvarO/PEP_301) was localized in the beginning of SD3 in a relatively conserved area of DBL1α (Figure S2). This region is semi-conserved and the DYVPQ**Y**LR/DYVPQ**F**LR is present in all DBL1α domains and is part of PoVL4 [36], which is conserved in-between DBL1α domains from different geographical origins (Figure S1). Although the antibody reactivity to these sequences might be a common feature in patients exposed to malaria, this epitope is only predicted to be partially surface exposed. Additional peptide-reactivity associated with rosette-disruption was found in NTS-DBL1α-3D7_var4_ (seven peptides) (Table 2). Four linear peptides were found within the NTS-DBL1α-IT4_var21_ protein. From the Ugandan sequences we also found two (UAS22 and UAS31) or one peptide (UAS29) associated with the ability to disrupt rosettes on FCR3S1.2.

**Table 2:** Peptides targeted by rosette disruptive antibodies in FCR3S1.2 iRBC.

Protein	Peptide	Sequence	Spearman r	p-value
It4var60	009	NEIELSARDVLENIG	0.3768	0.0281
It4var60	097	KRFDEGQKFECGNDK	0.5132	0.0019
It4var60	105	FECGNDKIIGNSDKY	0.5268	0.0014
It4var60	125	PRRRHICDQNLEFLD	0.5264	0.0014
It4var60	293	**SVPTNLDYVPQFLRW**	0.3902	0.0225
It4var9	009	TVNNLSATDVLEKIA	0.4101	0.0160
It4var9	013	LSATDVLEKIATGIY	0.3722	0.0302
It4var9	017	DVLEKIATGIYNQEK	0.3465	0.0447
It4var9	097	RFSNEGEAKCGSDKI	0.3438	0.0465
It4var9	293	**TNVPTNLDYVPQFLR**	0.4443	0.0085
TM284	065	NPCELKYQWHTNATR	0.4592	0.0063
TM284	245	EDWWNANRQEIWKAL	0.3705	0.0310
TM284	293	**PPTYFDYVPQYLRWF**	0.3775	0.0278
TM284	309	EWAEDFCTKRKHKLQ	0.4333	0.0105
TM284	376	IDNQQKEFEKQKKKY	0.3418	0.0479
varO	265	WKALTCSAPYYADYF	0.3879	0.0234
varO	301	**APPTYLDYVPQFLRW**	0.3625	0.0351
It4var21	021	EFGQKVHDEVHGEAK	0.3854	0.0244
It4var21	053	ETAFTVKSMQTESKY	0.3434	0.0468
It4var21	181	SFADIGDIIRGRDLY	0.3835	0.0252
It4var21	273	SSYFRATCSDTGQGP	0.5312	0.012
3D7var4	061	PFSAYRPNYGNPCEL	0.4328	0.0106
3D7var4	065	YRPNYGNPCELDYRF	0.436	0.0102
3D7var4	173	YTGYGIYKSGICTSL	0.4376	0.0096
3D7var4	301	**VLTNLDYVPQFLRWF**	0.3449	0.0457
3D7var4	309	**PQFLRWFEEWAEEFC**	0.4099	0.0160
3D7var4	341	SKKLYCSHNGYDCTK	0.5318	0.0012
3D7var4	345	YCSHNGYDCTKTIRN	0.5906	0.0002
C1388/UAS22	065	NTKLQTLTLHQVREY	0.4302	0.0111
C1388/UAS22	101	IFSKNIRNGNTILFD	0.3608	0.0361
C1413/UAS31	081	AWWKAHRDQVWKAIT	0.4200	0.0134
C1413/UAS31	101	KVDYFIKNSDGSRGF	0.3627	0.0350
C1429/UAS29	148	**DQVPTYFDYVPQYLR**	0.4214	0.0131

List of peptides, recognized on the peptide array, with a positive correlation with the ability of the plasma sample to disrupt FCR3S1.2 rosettes. Peptides indicated in bold are conserved between different parasite sequences and are further described in Figure S2.

## Discussion

Here we report on antibodies naturally acquired after infections with *P. faciparum*, their ability to recognize the surface of the iRBC and to disrupt rosettes of the rosetting model parasite FCR3S1.2. Further, by using a peptide array, we identify five antibody epitopes that correlate with rosette disrupting capacity. We also show that the presence of antibodies towards recombinant NTS-DBL1α is positively correlated with age (r = 0.2667 and p = 0.0023) and inversely correlated with parasitemia (r = −0.4943 and p<0.0001) in malaria-infected individuals. These results suggest that the presence of antibodies to this variable molecule is associated with number of infections, since in malaria endemic area individuals are infected repeatedly during their lifetime. Although the variation of the molecules expressed on the *P. falciparum* iRBC-surface is high, there is evidence that the parasite can induce cross-reactive antibodies. Previous studies showed that after a number of infections an individual in an endemic area develops immunity to the symptomatic disease [39],[40]. Those results encourage the search for antibodies that can cross-react in-between distinct malaria parasites.

At least part of the pathology caused by *P. falciparum* is due to the adherence of the parasite to endothelial cells and non-infected erythrocytes conferred by PfEMP1 [41]. The repertoire of PfEMP1 is vast although it seems to be restricted for the variants associated with severe disease [42],[43]. Severe malaria is associated with a particular subset of PfEMP1s that are encoded by group A *var* genes [37]. This group has two cysteines within the DBL1α domain, a feature of PfEMP1 associated with a rosetting phenotype. The fact that the repertoire of PfEMP1 associated with severe malaria appears to be somehow limited opens possibilities to target that group of molecules with a vaccine against severe malaria. It has long been known that naturally acquired antibodies have the ability to disrupt rosettes [44], but here we show that this capacity correlates with the presence of naturally acquired antibodies to NTS-DBL1α.

The ability to generate rosette disruptive antibodies is of advantage for the infected individual as formation of rosettes in the circulation obstructs the blood flow [45]. In a study conducted with samples from Benin, sera from children did not contain antibodies able to disrupt rosettes of the PAvarO model parasite [46]. In contrast with the Benin sample set, in our study we find a correlation between surface reactivity and rosette disruption activity. The parasite of this study, FCR3S1.2 and PAvarO [46] are both rosetting models and share some features characterizing their rosetting ability, such as sensitivity of rosettes to sulphated glycosaminogycans, the requirement of human serum for rosetting and a clear ABO blood group preference for rosette formation [13],[47]. Perhaps the most important difference between the two studies was the study area; while in Benin malaria is seasonal, with two distinct peaks, in Buea, our study area, malaria transmission takes place throughout the year. That might result in a higher number of annual infections, consequently resulting in higher diversity of acquired antibodies.

Since we found a correlation between the presence of specific antibodies to the NTS-DBL1α domain and the ability to disrupt rosettes of FCR3S1.2 we decided to dissect what those antibodies were targeting. Despite the limitations of the epitope mapping technique, such as inability to reproduce protein conformation, it was possible to map the B-cell repertoire to five peptides that correlated with recognition by rosette disruptive antibodies. Within the NTS-DBL1α domain from FCR3S1.2, the SD1 region carried most of the epitopes associated with the rosette disruption ability, whereas the major epitope found was C-terminal of SD1 and N-terminal of SD2. Interestingly, the SD1-SD2 region was previously shown to contain the heparin binding site for the rosetting parasite PAvarO [47]. Notably, those epitopes within the SD1–SD2 region were also predicted to be surface exposed in FCR3S1.2 iRBC, suggesting a possible involvement in binding to receptor. Indeed the lysine-residue number 97 (K97), part of one of the peptides hereby described, has recently been identified as possibly involved in heparin binding for It4var60 (Angeletti *et al.*, manuscript).

Recently we showed that immunization-induced rosette disruptive mouse antibodies predominantly target the SD3 region of the NTS-DBL1α-domain [12]. Here, only one peptide partially localized at the N-terminal end of the SD3 region was associated with the ability to disrupt rosettes of FCR3S1.2 parasites (PEP_293), while the majority of recognized epitopes localized in the SD1 region. The fact that we did not detect any other epitope within the SD3 region might be due to that the array is holding linear peptides with limited conformation; in addition the background reactivity of non-immune sera in this region was quite high, rendering the analysis more complex. Interestingly similar results have been obtained while analyzing natural responses to *P. vivax* Duffy binding protein (DBP) with majority of natural antibodies targeting regions in SD1 and SD2, while antibodies induced by immunization predominantly targeted SD3 [48]. Further, during human natural infections, the SD1 region might be more immunogenic, as compared to immunizations with recombinant antigen, or the conserved sequences in SD1 could be boosted upon subsequent or co- infections with distinct parasites.

Peptide PEP_293 of IT4_var60_ is localized at the SD2-SD3 boundary region and covers a conserved epitope which was earlier described as position of limited variability 4 (PoLV4) [49]. The PoLV4 motif consists of four amino acids, which are “PTNL” in the PfEMP1 encoded by IT4var60. Still, this part of the molecule is highly conserved between different DBL1α domain variants and is not predicted to be entirely surface exposed therefore probably not interacting with the red blood cell surface. From the molecular model of the NTS-DBL1α-domain it is interesting to note the structural proximity between the peptide PEP_293 identified herein and the RDSM motif, previously described as able to induce cross-reactive antibodies [32]. Both the peptides localize towards the end of SD2 and both seem to be able to be cross-recognized by antibodies, suggesting this region of the molecule to be an important target of strain transcending immunity in *P. falciparum*. While the function of anti-RDSM antibodies still remains elusive, here we show that presence of antibodies towards PEP_293 correlates with ability to disrupt rosettes.

The ability to disrupt rosettes of the FCR3S1.2 parasite strain was here used as a parameter to group the plasma samples into rosette disruptive (RD) or non-rosette disruptive (non-RD). It is known from previous studies, that the presence of rosette disrupting antibodies is considered a protective factor against severe disease [4],[40].

The antibody repertoire of the two groups (RD and non-RD) was analyzed on a peptide array that contained DBL domains from different *P. falciparum* parasite strains. Three of them were obtained from parasites with a rosetting phenotype (NTS-DBL1α-IT4_var9_, NTS-DBL1α-TM284S2 and NTS-DBL1α-PA_varO_); NTS-DBL1α-IT4_var9_, NTS-DBL1α-TM284S2, but not NTS-DBL1α-PA_varO_ harbored peptides recognized by antibodies with the ability to disrupt rosettes. Four out of five peptides found within NTS-DBL1α-IT4_var9_ are localized in the SD1 domain, mostly at the N-terminus of NTS sequence.

The fact that we found reactivity to peptides on the array associated with the rosette disruption capacity that cross-reacted with sequences derived from heterologous parasites might be explained by the fact that patients with uncomplicated malaria carry a broader antibody repertoire. Interestingly, of seven DBL1α domains analyzed we found peptides recognized at the C-terminus of the SD2 and N-terminus of the SD3 region. This common reactivity corresponds to the PoLV4 conserved region. Although the peptide DYVPQ**Y**LR/DYVPQ**F**LR was correlated with recognition by plasma from the RD group, this sequence is not entirely surface exposed and therefore it is not clear whether it could not mediate the binding to uninfected erythrocytes during rosetting. However, this peptide might be a marker of exposure to *P. falciparum*, as the plasma samples containing antibodies recognizing those peptides were also able to disrupt rosettes.

Our study suggests that naturally acquired antibodies that are able to disrupt rosettes target a number of different epitopes on the DBL1α domains of PfEMP1 encoded by IT4_var60_ with the majority located within SD1-SD2 region. These results should be taken into consideration in the design of a vaccine against severe malaria targeting the formation of rosettes.

## Materials and Methods

### Ethical approval

Informed consent was obtained from patients or their guardians. Ethical permissions for the study were obtained both in Cameroon and Sweden (numbers G379/900 and 2006/1323-31, respectively) and in China (number IRB#KMC20080001).

### Sample collection and study areas

The plasma collection was carried out in Buea, Cameroon as described before [34]. In Buea, malaria transmission takes place throughout the year but peaks during the rainy season that lasts from April to October. The entomological inoculation rate (EIR) has been shown to be approximately one infected *Anopheles* mosquito bite per person per night in this area during the rainy season.

Briefly, the study included *Plasmodium*-infected children between 6 months and 14 years of age that had a parasitemia above 10,000 iRBC/µl. The diagnosis was based on Giemsa-stained blood smears and clinical examination. Severe *P. falciparum* malaria was defined as a malaria infection requiring hospital admission and quinine or artemether infusion because of anemia, hyperparasitemia (parasitemia >5%), or severe symptoms including hyperpyrexia, seizures, prostration, and/or vomiting. Anemia was defined as hemoglobin less or equal to 55 g/l and hyperpyrexia as a body temperature over 41.5 C°. Cases with a positive blood smear for *P. falciparum* without complicating manifestations were classified as uncomplicated malaria and treated as outpatients with treatment *per os*. Blood was withdrawn from all patients with parasitemia above 10,000 iRBC/µl and collected in ethylene diamine tetraacetic acid (EDTA) tubes. Blood samples were centrifuged at 400×*g* for 5 min to separate RBCs, leukocytes, and plasma. The plasma was collected and stored at −70°C. Six samples from individuals living in low endemic area were also collected with the same procedure and used in the analysis. The samples were taken from Chinese individuals living in the low endemic region of Yunnan, close to the Burmese border.

### Parasite culture

Parasites were cultivated using standard methods [50]. Parasites were kept synchronous using 5% sorbitol (w/v). The parasitemia was counted and the rosetting rate was determined by calculating the number of trophozoite iRBCs forming rosettes, relative to the total number of trophozoite iRBCs present in the culture. A rosette was defined as at least two uninfected RBCs bound to one iRBC. The rosetting rate was maintained with Ficoll enrichment as previously described [50].

### Rosette disruption assay

The ability of patient plasma to disrupt rosettes was assayed as described before [4]. As a control a purified IgG fraction of a Malawian hyper-immune plasma pool was used [51]. Malaria naïve Swedish plasma was analyzed as negative control. Briefly, plasma was tested in 1∶5 dilution in 20 µl parasite culture, 5% hematocrit and 5% parasitemia. After incubating the samples at 37°C for 60 minutes, the parasites were stained with acridine orange and the rosettes were counted. For each sample at least 200 iRBC were analyzed.

### Flow cytometry assay on infected erythrocytes

Antibody binding to iRBC in trophozoite stage (24–30 hours post invasion) was tested using flow cytometry as previously described [13]. Briefly, the iRBCs were incubated with Cameroon patient plasma in dilution 1∶5 for 60 min at room temperature (RT). The iRBCs were washed trice with PBS/FCS 2% followed by 30 min incubation with a goat anti-human IgG antibody coupled to ALEXA488 (dilution 1∶100). For nuclear staining ethidium bromide was added at a final concentration of 2.5 µg/ml and the iRBCs were resuspended in PBS/FCS 2%. The cell count was done using flow cytometry (FACSCalibur, BD Bioscience, NJ USA) where 5000 iRBC were counted. The analysis was performed using the FlowJo software (Tree Star, Inc. Oregon, USA). All assays were done in duplicates. Reactivity against the iRBC was scored as mean fluorescence. The cut off value was determinate by the mean of the reactivity of 20 non-immune plasma samples (donors from Sweden, obtained from the blood bank) plus two standard deviation.

### Production of recombinant NTS-DBLα domains in *E.coli*

*E. coli* codon optimized NTS-DBL1α^It4var60^ domain of the parasite clone FCR3S1.2 were expressed as a C-terminal 6x histidine tagged recombinant proteins in the *E. coli* strain BL21 DE3 (ΔslyD) as described in [12]. Bacteria cultures were grown to OD_600_ = 0.8, then induced for 3 h at 37°C with 0.1 mM IPTG. Proteins were purified by Immobilized Metal Affinity Chromatography over Ni-NTA columns (Qiagen), eluted with 500 mM imidazole and further purified to homogeneity by size exclusion chromatography on a HiLoad 16/60 Superdex 75 pg colum (GE-Healthcare) [12].

### Enzyme-linked immunosorbent assay –ELISA with NTS-DBLα domains

Antibodies to the recombinant NTS-DBL1α^It4var60^ domain were analyzed by ELISA as described [52]. Briefly, maxisorp plates (Nunc, Roskild, Denmark) were coated with 0,2 µg of recombinant antigens over night at 4°C dissolved in 15 mM Na_2_CO_3_ and 35 mM NaHCO_3_ (pH 9.6). Plates were blocked with 1% bovine serum albumin (BSA) in PBS containing 0.1% Tween 20 (PBS-T) for 2 h at room temperature. Plasma samples (dilution: 1∶500) were added in duplicates and allowed to bind for 1 h at 37°C. Plates were washed five times with PBS-T and bound Immunoglobulin G (IgG) was detected by incubation for 1 h at 37°C with alkaline phosphatase–conjugated goat anti-human IgG (Sigma, St Louis, MO, USA) diluted 1∶1000 in PBS-T. The plates were washed and the assay developed by adding *p*-nitrophenyl phosphate (Sigma) as a substrate for 15 min. The optical density (OD) was read at 405 nm in an ELISA plate reader (Multiskan EX Version 1.0, Labsystems, Stockholm, Sweden). On each plate, a pool of plasma from 20 non-malaria exposed Swedish donors was assayed as well as control wells without plasma (background level). Cut off threshold for seropositivity was determined as the mean OD_405_ value from 20 individual non-immune Swedish donors plus two standard deviation.

### Peptide array

For detailed mapping of antibody binding sites, peptide-arrays of overlapping peptides were produced. The peptides were bound chemoselectively to the microarray surface by coupling an active amine of the peptide to an epoxy-group on the slide surface. The peptide microarrays were manufactured by JPT (JPT Peptide Technologies, Berlin, Germany) and each slide contained three identical sub-arrays. The arrays hold DBL-domains from long-term cultivated parasites as well as from Ugandan isolates (Table S2). The peptides are 15 amino acids long, with an overlap of 11 amino acids. The slides were incubated for 16 hours with human plasma (dilution: 1∶100) at 4°C in a humid chamber in PBS buffer containing 3% of FCS and 0.5% of Tween (T-PBS). Five washing steps followed the incubation step: two with T-PBS, followed by three washing steps with distillated water (all solutions were filtered). The slides were then incubated with 1∶500 goat anti-human Cy5 secondary antibody (Jackson ImmunoResearch) for two hours at room temperature, in a humid chamber, followed by washing steps. The microarrays slides were scanned at wavelength of 635 nm using a GenePix 4000B microarray scanner (Axon Instruments, CA, USA). The images were saved as TIFF and JPG format and analyzed using GenePixPro 7.0 software in combination with the.GAL file provided by JPT.

The image analysis was done as described at [53]. Quality control and data preprocessing were carried out as in [54]. Briefly, spots quality flags were assigned by GenePix and manually reviewed. The intensity data extracted from each subarray was saved as a GPR (GenePix result) file, and the median foreground and background intensities for the 635-nm wavelength from individual peptide spots were used in the analysis of the responses. All GPR files were imported into the R/BioConductor work environment [55]. Images from the individual subarrays were created and inspected in order to evaluate and exclude false positives from the analysis. For a measure of the strength of the response, we chose the ratio of median foreground to background (on a log scale). This response index was computed for all spots with a background greater than zero, and any spots with zero background were excluded. Any peptide with a high response on slides incubated only with buffer and the secondary Cy5-labeled was considered false positive and discarded for analysis. The array data was then normalized by using a linear model method as in [53]. The data for each of the four groups of slides were arranged in a large matrix, with columns identifying slide, subarray, and block, and all the analyses described below used these master data sets.

### Statistical Analysis

Principal Component Analysis (PCA) and Orthogonal Partial Least Squares (OPLS) from SIMCA package (Umetrics) were used to analyze multiple parameters. Values are expressed as mean ± SD. Comparisons between different groups were done by Mann-Whitney test for non-normally distributed samples. Spearman non-parametric correlation was used to correlate IgG levels and rosette disruption ability. Contingency table whereas samples were considered positive or negative for surface recognition and rosetting disruption followed by Fischer's exact test was applied using GraphPad Prism version 5.0 (GraphPad Software, CA, US).

### Protein modeling

The 3D structure of NTS-DBL1α It4var60 was modeled using the Phyre2 server. Structural visualizations and high-resolution images were generated using PyMol (The PyMOL Molecular Graphics System, Version 1.3, Schrödinger, LLC.).

### Prediction of B cell epitopes

Prediction of B cell epitopes was carried out by submitting the protein sequences to Bepipred server [35]. Cut off for positivity was set to 0.5.

## Supporting Information

Figure S1 **Alignment of NTS-DBL1α sequences and their reactivity on peptide array.** Peptides covering NTS-DBL1α sequences from different parasite lines were tested for reactivity with rosette disruptive or non-disruptive plasma samples. Peptides that correlated with the ability to disrupt rosettes on FCR3S1.2 parasites are colored in red. The sequences on the highlighted box correspond to the SD2–SD3 region.
(PDF)

Figure S2 **Correlation of SD2–SD3 immune recognition and the ability to disrupt rosettes on FCR3S1.2.** Sequences at the C-terminus of SD2 and N-terminus of SD3 of six different DBL1α were correlated with the ability to disrupt rosettes on FCR3S1.2.
(PDF)

Table S1 **Experimental and clinical parameters of the plasma samples included in the study.** Summary of parameters of the 74 plasma samples analyzed in the study. RD: ability of the sample to disrupt FCR3S1.2 rosettes (%); MFI: recognition of the FCR3S1.2 iRBC surface by flow cytometry; It4var60 reactivity: ELISA reactivity towards the recombinant NTS-DBL1αIt4var60 domain.
(PDF)

Table S2 **List of proteins included in the peptide array.** PfEMP1 domains analyzed in the array are fragmented in overlapping peptides whereas each peptide is 15 amino acids long overlapping by 11 amino acids.
(PDF)
