# Supplementary material for: B-Cell Epitopes in NTS-DBL1α of PfEMP1 Recognized by Human Antibodies in Rosetting Plasmodium falciparum
Source: PLoS One. 2014 Dec 1;9(12):e113248. doi: 10.1371/journal.pone.0113248 (PMC4249881; doi:10.1371/journal.pone.0113248)
Supplement: Figure S1 — Alignment of NTS-DBL1α sequences and their reactivity on peptide array. Peptides covering NTS-DBL1α sequences from different parasite lines were tested for reactivity with rosette disruptive or non-disruptive plasma samples. Peptides that correlated with the ability to disrupt rosettes on FCR3S1.2 parasites are colored in red. The sequences on the highlighted box correspond to the SD2–SD3 region. (PDF) [file pone.0113248.s001.pdf]

Supporting Information

Figure S1 – Alignment of NTS-DBL1α sequences and their reactivity on peptide array. Peptides covering NTS-DBL1α sequences from different parasite lines were tested for reactivity with rosette disruptive or non-disruptive plasma samples. Peptides that correlated with the ability to disrupt rosettes on FCR3S1.2 parasites are colored in red. The sequences on the highlighted box correspond to the SD2-SD3 region.

```
It4var60          -MAP-------KGRSTNEIELSARDVLENIGIGIYNQEKI-KKNPYEQQLKGTLSNARFHDGLHKAADLGVIPGPSHFSQ   71
PAvarO            -MGSSHSTNDTKSPTLSESHKSARNVLENIGIKIYNQEIK-KKNPYEQQLKGTLSRAQFVDALSSRYGY-VRNSDGNSCN   77
It4var9           -MTP-------KRTSRTVNNLSATDVLEKIATGIYNQEKE-KVYPYENELKGILSNAIFVDQLRKELNI-ESPGPSDSCS   70
UAS31             --------------------------------------------------------------------------------
3D7var4_PFD1235w  MGNASSSEGEAKTPSLTESHNSARNILEGYAESIKEQASK-DAKIHGHHLKGDLAKAVFRHPFS-----AYRPNYGNPCE   74
It4var21          --------MATSGGSGGTQDEDAKHVLDEFGQKVH-DEVHGEAKNYVSELKGSLSLASILGET-------AFTVKSMQTE   64
UAS29             --------------------------------------------------------------------------------
TM284S2           ------MAPQKAAAPDYSSAKDAKELLDMIGEEVYKEKVKNAANDFREKLKGTLSQATFEEAPK------EQQTPGNPCE   68
UAS22             --------------------------------------------------------------------------------

It4var60          LYYKKHTNNTKYYKDDRHPCHGRQGKRFDEGQKFECGNDKIIGNSDKYG--SCAPPRRRHICDQNLEFLDN-NHTDTIHD   148
PAvarO            LDHLFHTNIKTGYNEGRKPCYGREQNRFDENAEAYCNSDKIRGNENNSNGTACAPPRRRHICDQNLEFLDN-KNTNTTHD   156
It4var9           LDHKFHTNINTEYTEGRKPCYERNEKRFSNEGEAKCGSDKIRDYGIKSAGGACAPFRRQNLCDRNLEYLIN-KNTNTTHD   149
UAS31             --------------------------------------------------------------------------------
3D7var4_PFD1235w  LDYRFHTNVWHRNAEDRNPCLFSRAKRFSNEGEAECNGGIITGNKGECG--ACAPYRRRHICDYNLHHINE-NNIRNTHD   151
It4var21          SKYTELIEANSKRNPCKKDGKGNDVDRFSVKEQAGYDNKKMKCSNGD----ACAPFRRLHLCNKNFPNMNSYDSSKAKHD   140
UAS29             --------------------------------------------------------------------------------
TM284S2           LKYQWHTNATRGKNYPCRTG---TEKRFSEVSGGECDEKKIKDNKGKEG--ACAPYRRLHLCVRNLENISALDKIN-NDT   142
UAS22             --------------------------------------------------------------------------------

                                                   :*. * ******.**::* *:    .             : . :.
It4var60          VLGNVLVTAKYEGESIVNDHPDKKNNGNKS--GICTSLARSFADIGDIVRGRDMFKPND----------KDAVRHGLKVV   216
PAvarO            LLGNVLVTAKYEGNYIVNDHPDKNSNGNKS--GICTSLARSFADIGDIVRGRDMFLPNK----------DDKVQKGLQVV   224
It4var9           LLGNVLVTAKYEGDSIVNNHPDKNSSGNKS--SICTALARSFADIGDIVRGRDMFKPND----------ADKVEKGLQVV   217
UAS31             -------------DYIVQNHPNKDTS------EVCTALARSFADIGDIVKGKDMFKRTD----------NDEVWKGLRAV   51
3D7var4_PFD1235w  LLGNLLVMARSEGESIVKSHEYTGYGIYKS--GICTSLARSFADIGDIIRGKDLYRRDSR---------TDKLEENLRKI   220
It4var21          LLAEVCMAAKYEGESIKTHYPKYDSKYPGSDFPMCTMLARSFADIGDIIRGRDLYLGNKKKKQNGKETEREKLEQKLKEI   220
UAS29             -------------ESLIHYHEQYQNKYGDS--QLCTMLARSFADIGDIVRGKDLYRGNDEEKK-----QRDKLDKNFKKY   60
TM284S2           LLADVCLAALHEGAAISADHGQYQQTNDSS--QLCTMLARSFADIGDIIRGKDLYRGNN---------GKDKLEENLKKI   211
UAS22             ---ESLVKKYKEYK---EKNPNFN---------ICTALERSFADIGDIIRGKDLHLRHEPG--------IQHLEKRLESM   57

                  * :* .                     .  ::** **  :*. :* .:*                              *
It4var60          FKKIYDKLSP----KVQEHYKDVDGSGNYYKLREDWWTANRDQVWKAITYKAP-QDANYFR--NVSGTT--MAFTSAGKC   287
PAvarO            FKKIYKSLTP----EARKHYAHGDGSGNYSKLREDWWTINREQIWKALTCSAP-YYADYFR--KGSDGT--LHFSSHGKC   295
It4var9           FGKIYNSLPS----PAQKHYAHDDGSGNYYKLREDWWAINRKEVWKAITCRAP-NEANFFR--NISGNM--KAFTSQGYC   288
UAS31             FGKIYKSLPS----PAQNYYA-DDGSGNYYKLREAWWKAHRDQVWKAITCKAP-PKVDYFI--KNSDGS--RGFTSQGQC   121
3D7var4_PFD1235w  FANIYKELKNGKKWAEAKEYYQDDGTGNYYKLREAWWALNRKDVWKALTCSAP-RDAQYFI--KSSVRD--QTFSN-DYC   294
It4var21          FKKIHDNLKDKE----AQKRYNGDEDPNFYKLREDWWTANRETVWGAMTCSKELDASSYFR--ATCSDTGQGPSQTHNKC   294
UAS29             FQQIHDDVMSTKGRHGVKARYKADKNNNFFQLREDWWALNRETVWEALTCKAD-NSNRYFR--QTCNDN-ESLSHASHKC   136
TM284S2           FGNIYKDVTKGGKNVDALKTRYEDATGNYYKLREDWWNANRQEIWKALTCDAPNGDVHYFR--KTCSMG---QSHVNDKC   286
UAS22             FQNIQN--------NNTKLQT-----LTLHQVREYWWALNRKEVWKAITCGATMNDIFSKN-----IRNG-NTILFDYKC   118

                                        * :*****:**
It4var60          RH----------------NDNSVPTNLDYVPQFLRWYDEWADDFCRIRNHKLQKVKDTCQGYNNSGYRIYCSGDGEDCTN   351
PAvarO            GH----------------NEGAPPTYLDYVPQFLRWFEEWSEEFCRIKKIKIDKVKKECRDEQN---KKYCSGDGHDCTQ   356
It4var9           GH----------------SETNVPTNLDYVPQFLRWFDEWAEEFCRIRKIKLENVKKECRDEPN---NKYCSGDGHDCKR   349
UAS31             GR----------------NEINVPTNLDYVPQFLR---------------------------------------------   140
3D7var4_PFD1235w  GH----------------GEHEVLTNLDYVPQFLRWFEEWAEEFCRIKKIKLGKVKEACRDDSK---KLYCSHNGYDCTK   355
It4var21          RCDKDKGANAGKPKAGDGDVTIVPTYFDYVPQYLRWFEEWAEDFCRKKKKKLENLEKQCRGKDKSDEYRYCSRNGYDCEQ   374
UAS29             RC---------RSKNFKEETDQVPTYFDYVPQYLR---------------------------------------------   162
TM284S2           RC-------------LNGDP---PTYFDYVPQYLRWFEEWAEDFCTKRKHKLQNAIKICRGDSGND--RYCDLNGYDCTK   348
UAS22             AH---------------HVNKDVPTNLDYVPQFLR---------------------------------------------   138

It4var60          ILKQNFNIVSDFFCPSCKTECTNYKKWINKKQGEFNKQKKKY--------------------------------------   393
PAvarO            TNLSHNQIFVDLDCPRCQDQCIKYNEWIVKKLEEFYKQNLKY--------------------------------------   398
It4var9           TYLKDNTIFIDLNCPRCENACSNYTKWIEIQRKQFDKQKRKY--------------------------------------   391
UAS31             --------------------------------------------------------------------------------   140
3D7var4_PFD1235w  TIRNKDILSDNPKCTGCSVKCKVYELWLRNQRNEFEKQKKKYYK------------------------------------   399
It4var21          TISRKGKVRMGKGCTDCFFACHSYENWIDNQRKQFDKQKKY---------------------------------------   415
UAS29             --------------------------------------------------------------------------------   162
TM284S2           TARGKNKRFSNDECYKCSLPCDHFVPWIDNQQKEFEKQKKKY--------------------------------------   390
UAS22             --------------------------------------------------------------------------------   138
```
