# Supplementary material for: B-Cell Epitopes in NTS-DBL1α of PfEMP1 Recognized by Human Antibodies in Rosetting Plasmodium falciparum
Source: PLoS One. 2014 Dec 1;9(12):e113248. doi: 10.1371/journal.pone.0113248 (PMC4249881; doi:10.1371/journal.pone.0113248)

Supporting Information

Figure S2 - Correlation of SD2-SD3 immune recognition and the ability to disrupt rosettes on FCR3S1.2. Sequences at the C-terminus of SD2 and N-terminus of SD3 of six different DBL1α were correlated with the ability to disrupt rosettes on FCR3S1.2.

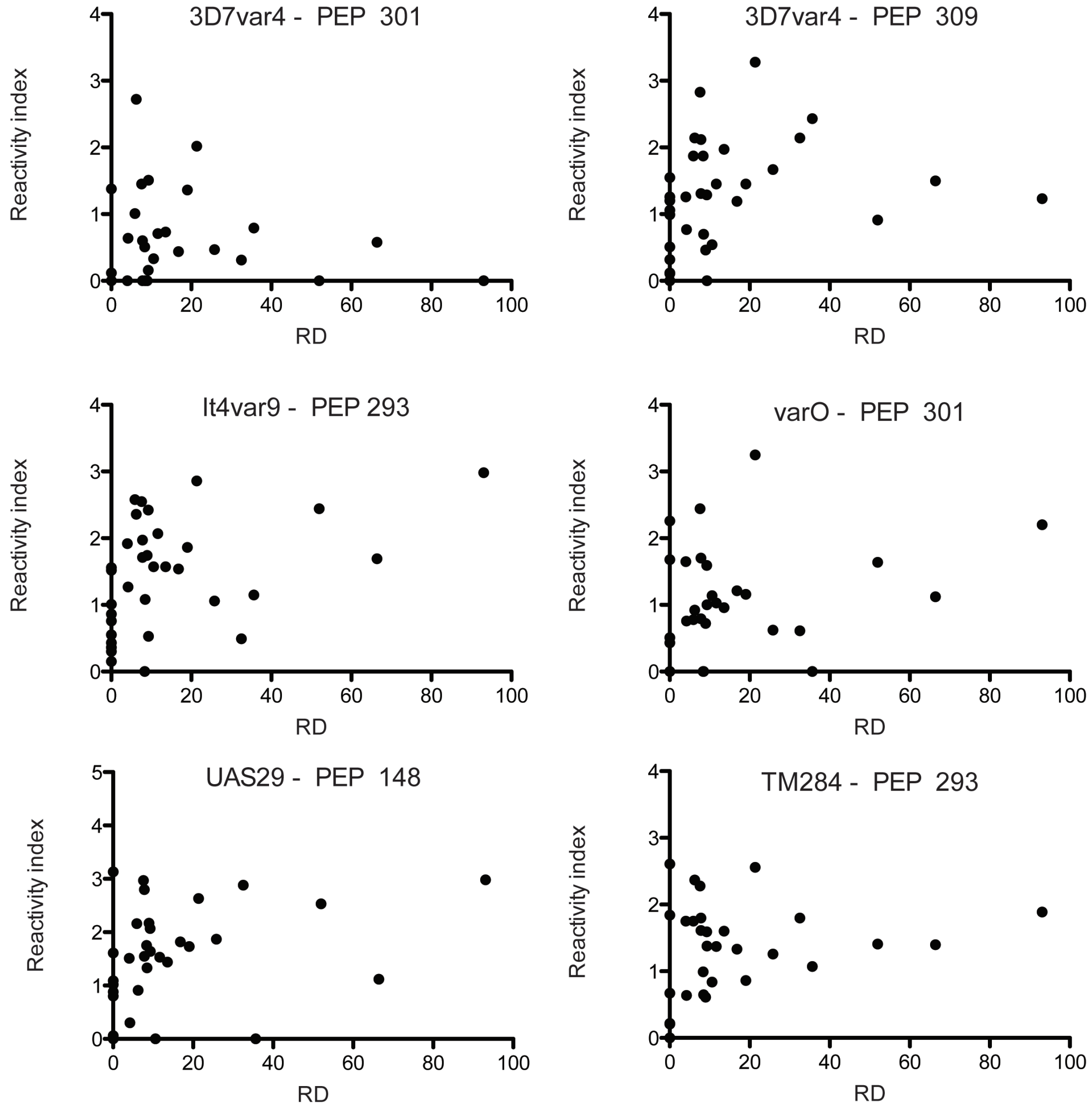

Supplement: Figure S2 — Correlation of SD2–SD3 immune recognition and the ability to disrupt rosettes on FCR3S1.2. Sequences at the C-terminus of SD2 and N-terminus of SD3 of six different DBL1α were correlated with the ability to disrupt rosettes on FCR3S1.2. (PDF) [file pone.0113248.s002.pdf]
