# Supplementary material for: B-Cell Epitopes in NTS-DBL1α of PfEMP1 Recognized by Human Antibodies in Rosetting Plasmodium falciparum
Source: PLoS One. 2014 Dec 1;9(12):e113248. doi: 10.1371/journal.pone.0113248 (PMC4249881; doi:10.1371/journal.pone.0113248)
Supplement: Table S1 — Experimental and clinical parameters of the plasma samples included in the study. Summary of parameters of the 74 plasma samples analyzed in the study. RD: ability of the sample to disrupt FCR3S1.2 rosettes (%); MFI: recognition of the FCR3S1.2 iRBC surface by flow cytometry; It4var60 reactivity: ELISA reactivity towards the recombinant NTS-DBL1αIt4var60 domain. (PDF) [file pone.0113248.s003.pdf]

Supporting Information

Table S1 – Experimental and clinical parameters of the plasma samples included in the study. Summary of parameters of the 74 plasma samples analyzed in the study. RD: ability of the sample to disrupt FCR3S1.2 rosettes (%); MFI: recognition of the FCR3S1.2 iRBC surface by flow cytometry; It4var60 reactivity: ELISA reactivity towards the recombinant NTS-DBL1α^{It4var60} domain.

Isolates	RD	MFI	It4var60 reactivity	Age (months)	Breath	Pulse	Temperature 0º.C	Parasites/uL	Glucose (mmol/L)	Hemoglobin (g/L)	Blood Group
CBM001	0,00		1,16	16	40	112	37,9	80	7,9	104	A
CBM002	0,00		0,81	55	40	120	37,6	120	7,2	98	A
CBM003	0,00		1,46	53	40	100	37,9	120	8,6	90	O
CBM004	12,68	164,035	0,65	49	30	120	37,7	256000	6,2	76	O
CBSr005	0,00		0,50	47	40	140	37,6	2000	5,2	92	O
CBM006	2,73	7,48	0,62	36	36	120	37,5	200	8,2	104	A
CBM007	37,17	160,45	1,17	55	32	100	37,5	120	9,1	105	O
CBSa008	0,00	88,125	0,96	10	32	140	37,9	6800	67	29	AB
CBM009	10,16		0,75	84	26	90	36,8	1000	8,4	109	O
CBM010	41,75		1,40	73	27	100	37,1	400	9,6	113	O
CBM011	41,46		1,56	106	28	100	37,6	4080	8,7	126	O
CBM012	23,92		1,03	90	32	100	37,3	1840	7,7	114	A
CBM013	50,90		1,04	91	28	120	40,3	6000	7,5	99	B
CBM014	5,53	136,08	1,37	7	40	140	37,9	1440	8,3	83	O
CBM015	1,06		1,66	51	40	100	37,5	400	7,3	97	A
CBM020	10,65	30,6	1,47	50	36	100	37,1	12000	8,1	106	O
CBM021	9,32		0,91	117	28	100	37,8	41680	7,1	113	O
CBM022	5,57	179,635	0,61	20	40	120	37,7	11080	8,5	74	O
CBM023	2,31	91,525	0,43	84	32	120	38,1	15600	9,6	83	AB
CBM024	0,00	101,97	1,40	66	32	104	37,3	23250	9,6	83	O
CBM028	4,16	146,79	1,22	72	28	120	37,6	14086	8,8	85	O
CBM029	6,19	109,46	0,54	64	32	128	36,8	72000	8,2	100	A
CBM031	8,99	109,725	1,25	152	36	100	37,6	17300	8,2	68	A
CBM032	27,50	164,505	1,53	96	24	80	37,8	10000	7,4	108	O
CBM033	7,65	153,395	0,34	68	28	108	37,6	320000	7,9	121	O
CBSa035	7,78	111,45	1,20	22	66	88	39,2			39	A
CBM036	93,08	306,13	1,73	60	28	100	37,6	9500	8,2	98	O
CBM037	7,60	33,375	0,84	50	34	110	38,8	14640	10,1	94	A
CBM040	2,69	108,575	0,56	36	56	120	40,2	210000	8,3	105	O
CBM041	51,97	352,39	1,30	119	32	84	37,3	10820	7,8	120	A

CBM042	45,37		0,63	21	36	108	37,3	22440	8,4	90	O
CBM043	20,35	283,81	1,07	41	40	120	37,7	11080	7,5	101	O
CBM045	11,15		0,82	41	40	120	37,5	84920	9,6	124	O
CBM046	9,26	189,515	0,77	59	32	100	37,5	10900	7,5	101	B
CBSa047	7,80	6,5	0,75	88	40	140	40,4	27850	8,1	55	O
CBM048	11,74	155,595	1,00	10	40	132	37,6		10,6	85	O
CBSa053	0,00		0,36	36	24	83	40			51	O
CBM054	16,79	222	1,09	59	44	120	40	10300	8,1	94	B
CBM056	0,00	233,945	0,45	35	44	160	38,9	27300	8,6	75	A
CBSr060	7,59	12,7	0,76	62	30	120	38,5			96	B
CBM063	0,00	106,845	1,15	11	36	136	38,9	294000	9,3	86	O
CBM064	7,48	89,775	0,51	81	32	140	39,2	11200	7,9	121	O
CBSa065	31,08		0,46	89	28	136	39,5	25060	6,7	50	O
CBM066	62,75		0,78	30	40	112	37,6	145000	7,3	74	A
CBM068	66,44	336,45	1,45	27	40	120	37,3	10900	7,8	94	B
CBM069	0,00		0,29	63	24	112	37,2	39200	8,8	75	A
CBM072	53,30	206,61	0,58	86	25	135	40,5	34180	9,2	92	B
CBM073	14,34		1,02	42	30	140	38	16400	8,2	102	O
CBM074	22,25	194,95	0,54	24	25	130	40,8	34900	7,6	65	B
CBM075	10,57	185,385	1,22	26	25	180	40,5		6,1	109	AB
CBM077	0,62	87,06	1,03	62	28	100	40			109	A
CBM078	8,09		0,49	27	44	100	36,5	96000	8,1	89	A
CBM079	28,58		0,43	60	40	120	38,8	57300	7,1	109	A
CMB080	4,42	142,025	1,11	63	40	120	39,4	282660	6	96	O
CBM081	26,97	163,685	0,94	172	36	128	38,5	19250	7,6	107	A
CBM083	6,11	133,15	0,65	51	42	110	38	42100	8,6	84	B
CBM084	0,00	80,11	0,63	32	40	80	38,5	155000	11,5	105	B
CBM086	0,58	150,27	1,01	60	30	86	38,1	14780	6,6	100	O
CBSa088	11,63	119,08	0,48	35	30	160	37,5			47	O
CBSa089	4,20	4,4	0,95	25	20	120	39			52	O
CBSa091	8,45	5,5	0,44	19	20	100	38			51	O
CBM138	4,29	17,045	0,66	24	-	-	37,5				B
CBM139	3,05	109,62	0,28	24	30	80	37,6	6700		76	O
CBSc155	25,83	5,495	1,07	84	30	88	38,8			102	A
CBSa162	5,94	85,62	1,24	20	30	140	40			29	B
CBSa168	19,02	8,895	1,08	13			39,3			31	O
CBCs169	12,51		0,32	36			37,6	24000		63	O
CBM170	6,35		0,33	156			38,6			113	A
CBSa171	9,39		0,87	36	24	58	36,4			50	O
CBM172	16,09		0,47							112	AB
CBSa173	8,52		0,75	36	30	130	38,5			36	A

CBM174	0,00		0,35	48	30	98	38,5			57	O
CBSa175	2,58		0,39	20			37,9			45	B
CBSr176	7,21		0,89	110	20	80	40,2			98	O
