# Supplementary material for: B-Cell Epitopes in NTS-DBL1α of PfEMP1 Recognized by Human Antibodies in Rosetting Plasmodium falciparum
Source: PLoS One. 2014 Dec 1;9(12):e113248. doi: 10.1371/journal.pone.0113248 (PMC4249881; doi:10.1371/journal.pone.0113248)
Supplement: Table S2 — List of proteins included in the peptide array. PfEMP1 domains analyzed in the array are fragmented in overlapping peptides whereas each peptide is 15 amino acids long overlapping by 11 amino acids. (PDF) [file pone.0113248.s004.pdf]

Supporting Information

Table S2 - List of proteins included in the peptide array. PfEMP1 domains analyzed in the array are fragmented in overlapping peptides whereas each peptide is 15 amino acids long overlapping by 11 amino acids.

Protein - Parasite	Domain	Peptides (N)
PfEMP1 - FCR3S1.2 (It4var60)	NTS-DBL1a	96
PfEMP1 – R29 (It4var9)	NTS-DBL1a	95
PfEMP1 - VarO	NTS-DBL1a	97
PfEMP1 – TM284S2	NTS-DBL1a	95
PfEMP1 - FCR3S1.2 (It4var21)	NTS-DBL1a	101
PfEMP1 - 3D7var4 - 3D7	NTS-DBL1a	97
PfEMP1 - UAS22	DBL1a	32
PfEMP1 - UAS29	DBL1a	33
PfEMP1 - UAS31	DBL1a	38
